# Supplementary material for: Chromothripsis during telomere crisis is independent of NHEJ, and consistent with a replicative origin
Source: Genome Res. 2019 May;29(5):737–49. doi: 10.1101/gr.240705.118 (PMC6499312; doi:10.1101/gr.240705.118)
Supplement: Supplemental Material [file supp_gr.240705.118_Supplemental_file_1.zip › contigs/annotated_contigs/DB93/contig.2.DB93_length_435_mean_cov_9.55632183908.docx]

**DB93_length_435_mean_cov_9.55632183908**

```
GCTAATTTTTTGGTATTTTTTTGTAGAGATGGGGTCTCATCACATTGCCCAGGCTGGTTCTGACCTCCTGGGCTCAAGTGATCCACCCA
 >chr17:50313923-50314231 + E=5e-168 p=1e-02
CTTCAGCCTCCCAAAGTGCTGGGATTACAGGCGTGAGCCATCATTCTGGGCCTAAGTAACCCTATCATCTAAATATGCCTCTTAGGAAA

TTGCATCATAGTCTTCAAGTGGAAGAGGCACGTTATTTAGAGTATATTGACTAAGAGTGCATTTTCTGGCCTCAGGAAAAAAAAGGTTT

AAATCCTGGTGTTATCAATGATTAGCCATGGGACCTTGT|ATCCCAGGATTTCCCTAGTTGAGACCTCCAGTGTCATTTTAAGGAGGCT
                                         >chr17:47900289-47900384 - E=1e-45
GACTCTGGGCTCCTGACAGCCACAGAGTAGGGCAAGAAGTTTAAGA|TCGGAAGAGCGTCGTGTAGGGAAAGAGTGTTCAG
```
